# A Smoothed Version of the Lassosum Penalty for Fitting Integrated Risk Models Using Summary Statistics or Individual-Level Data

**DOI:** 10.3390/genes13010112

**Published:** 2022-01-06

**Authors:** Georg Hahn, Dmitry Prokopenko, Sharon M. Lutz, Kristina Mullin, Rudolph E. Tanzi, Michael H. Cho, Edwin K. Silverman, Christoph Lange, on the behalf of the NHLBI Trans-Omics for Precision Medicine (TOPMed) Consortium

**Affiliations:** 1 Harvard T.H. Chan School of Public Health, Harvard University, 677 Huntington Ave, Boston, MA 02115, USA; sharon.lutz@channing.harvard.edu (S.M.L.); clange@hsph.harvard.edu (C.L.); 2 Genetics and Aging Research Unit, McCance Center for Brain Health, Department of Neurology, Massachusetts General Hospital, Boston, MA 02114, USA; dprokopenko@mgh.harvard.edu (D.P.); kmullin1@mgh.harvard.edu (K.M.); tanzi@helix.mgh.harvard.edu (R.E.T.); 3 Department of Medicine, Brigham and Women’s Hospital, Harvard University, Boston, MA 02115, USA; remhc@channing.harvard.edu (M.H.C.); reeks@channing.harvard.edu (E.K.S.)

**Keywords:** integrated risk model, lassosum, nesterov, polygenic risk scores, smoothing

## Abstract

Polygenic risk scores are a popular means to predict the disease risk or disease susceptibility of an individual based on its genotype information. When adding other important epidemiological covariates such as age or sex, we speak of an integrated risk model. Methodological advances for fitting more accurate integrated risk models are of immediate importance to improve the precision of risk prediction, thereby potentially identifying patients at high risk early on when they are still able to benefit from preventive steps/interventions targeted at increasing their odds of survival, or at reducing their chance of getting a disease in the first place. This article proposes a smoothed version of the “Lassosum” penalty used to fit polygenic risk scores and integrated risk models using either summary statistics or raw data. The smoothing allows one to obtain explicit gradients everywhere for efficient minimization of the Lassosum objective function while guaranteeing bounds on the accuracy of the fit. An experimental section on both Alzheimer’s disease and COPD (chronic obstructive pulmonary disease) demonstrates the increased accuracy of the proposed smoothed Lassosum penalty compared to the original Lassosum algorithm (for the datasets under consideration), allowing it to draw equal with state-of-the-art methodology such as LDpred2 when evaluated via the AUC (area under the ROC curve) metric.

## 1. Introduction

Polygenic risk scores are a statistical aggregate of risks typically associated with a set of established DNA variants. If only genotype information of an individual is used to predict its risk, we speak of a polygenic risk score. A polygenic risk score with added epidemiological covariates (such as age or sex) is called an integrated risk model [1]. The goal of both polygenic risk scores and integrated risk models is to predict the disease risk of an individual, that is the susceptibility to a certain disease. Such scores are usually calibrated on large genome-wide association studies (GWAS) via high-dimensional regression of a fixed set of genetic variants (and additional covariates in case of an integrated risk model) to the outcome. In this article, we focus on the more general case of an integrated risk model.

As the potential for broad-scale clinical use to identify people at high risk for certain diseases has been demonstrated [2], polygenic risk scores and integrated risk models have become widespread tools for the early identification of patients who are at high risk for a certain disease and who could benefit from intervention measures [3,4,5]. However, the accuracy of current polygenic risk scores, measured with the AUC metric (area under the ROC Curve, where ROC stands for receiver operating characteristic, see in [6]), varies substantially across application areas. For instance, the AUC achieved by state-of-the-art methods ranges from around 0.8 for type 1 diabetes to around 0.7 for coronary artery disease and schizophrenia [7], while for atrial fibrillation the AUC is around 0.64 [8], a value which is considered less than acceptable [6,9]. For this reason, increasing the accuracy of scores is desirable, which is the focus of the proposed smoothing approach.

One popular way to fit a polygenic risk score is the “Lassosum” approach of the authors of [7]. Note that in [7], no integrated risk models are considered. The Lassosum method is based on a reformulation of the linear regression problem y=Xβ+ϵ, where $X\in R^{n\times p}$ denotes SNP data for *n* individuals and *p* SNP locations, $y\in R^{n}$ denotes a vector of outcomes, $\beta \in R^{p}$ is unknown, and $\epsilon ∼N_{n}\left(0,\sigma^{2}I_{n}\right)$ is an *n*-dimensional, independently and normally distributed error term with mean zero and some variance $\sigma^{2}>0$ (where $I_{n}$ denotes the *n*-dimensional identity matrix). The authors start with the classic Lasso objective function $L\left(\beta \right)={∥y-X\beta ∥}_{2}+2\lambda {∥\beta ∥}_{1}$, where λ≥0 denotes the Lasso regularization parameter controlling the sparseness of the solution, and rewrite it using the SNP-wise correlation $r=X^{⊤}y$ as

(1) $$\begin{array}{c} L\left(\beta \right)=y^{⊤}y+\left(1-s\right)\beta^{⊤}X_{r}^{⊤}X_{r}\beta -2\beta^{⊤}r+s\beta^{⊤}\beta +2\lambda {∥\beta ∥}_{1}, \end{array}$$

where $X_{r}$ denotes the matrix of genotype data used to derive estimates of LD (linkage disequilibrium), λ≥0 is the Lasso regularization parameter controlling the sparseness of the estimate, and s∈(0,1) is an additional regularization parameter used to ensure stability and uniqueness of the Lasso solution. As in [7], we assume in this article that estimates of the correlations r can be obtained from publicly available summary statistics databases, and that estimates of the LD matrix $X_{r}^{⊤}X_{r}$ are obtained from publicly available genotype databases (such as the 1000 Genomes Project). However, the Lassosum objective function can also be used to compute a polygenic risk score using raw data. Importantly, in [7] the authors derive an iterative scheme to carry out the minimization of Equation (1) which only requires one column of $X_{r}$ at a time, thus avoiding the costly computation of the matrix $X_{r}^{⊤}X_{r}\in R^{p\times p}$.

In this work, we consider a different approach for minimizing Equation (1). Using the methodology in [10], we propose to smooth the non-differentiable $L_{1}$ penalty in Equation (1), thus allowing us to compute explicit gradients of Equation (1) everywhere. This in turn allows us to efficiently minimize the Lassosum objective function using a quasi-Newton minimization algorithm such as BFGS (Broyden–Fletcher–Goldfarb–Shanno). Besides enabling a more efficient and more accurate computation of the score, our work extends the one of [7] in that we do not solely consider polygenic risk scores, but the more general integrated risk models. The proposed smoothed Lassosum can be applied to either summary statistics (when using *X* and r as previously described), as well as individual-level data (when using *X* and y directly in either the Lasso or Lassosum objective function; in the latter case, y is converted into “correlations” r via $r=X^{⊤}y$).

Our approach follows as a special case from in [11,12], who propose a general framework to smooth $L_{1}$ penalties in a linear regression. Importantly, employing a smoothing approach has a variety of theoretical advantages following directly from in [11]. Apart from obtaining explicit gradients for fast and efficient minimization, the smoothed objective is convex, thus ensuring efficient minimization, and it is guaranteed that the solution (the fitted integrated risk model) obtained by solving the smoothed Lassosum objective is never further away than a user-specified quantity from the original (unsmoothed) objective of [7].

We evaluate all aforementioned approaches by computing an integrated risk model in two experimental studies, one on Alzheimer’s disease using the summary statistics of [13,14], and one on COPD (chronic obstructive pulmonary disease) using individual-level spirometry data [15]. In the first case, the endpoint is binary, whereas in the second study the endpoint is continuous. Our experiments demonstrate that smoothing the Lassosum objective function results in a considerably enhanced performance of the Lassosum approach for the datasets we consider, allowing it to draw equal with approaches such as LDpred2 [16] or PRScs [17].

Analogously to the original Lasso of [18], the $L_{1}$ penalty employed in Equation (1) causes some entries of $\arg\min_{\beta \in R^{p}}L\left(\beta \right)$ to be shrunk to zero exactly (provided the regularization parameter λ is not too small). Therefore, Lassosum performs fitting of the polygenic risk score or integrated risk model and variable selection simultaneously.

This article is structured as follows. A literature review is given in Section 1.1. Section 2 introduces the smoothed Lassosum objective function and discusses its minimization, the theoretical guarantees it comes with, and its drawbacks. Section 3 evaluates the proposed approach, the original Lassosum approach, as well as additional state-of-the-art methods in two experimental studies on both Alzheimer’s disease and COPD. The article closes with a discussion in Section 4, and some final remarks are given in the conclusions of Section 5. The appendix contains two figures showing plots of principal components for the genotype dataset employed in Section 3.1.

The methodology of this article is implemented in the R package *smoothedLasso* (see the function *prsLasso* in the package), available on CRAN [19].

### 1.1. Literature Review

Several methodological approaches have been considered in the literature to compute a polygenic risk score or an integrated risk model for a given population [20], and to predict a given outcome (disease status).

A simple way to calculate a polygenic risk score is to threshold *p*-values coming from GWAS summary statistics. If all genetic markers are used, we speak of an unadjusted polygenic risk score. However, if SNPs in linkage disequilibrium (LD) with each other are included in the score, their contribution will be exaggerated, thus making informed LD-pruning of single nucleotide polymorphisms (SNPs) in LD necessary [21]. The selective removal of less significantly related SNPs to reduce LD is called LD-clumping [22]. Such approaches are computationally simple and fast, but have limited accuracy [23]. However, the (optimal) choice of the threshold is an issue, as this determines the number of SNPs to be included [22]. As a result, scores are often constructed for a variety of thresholds [7,24].

Accuracy can be increased by incorporating GWAS summary statistics via Bayesian methods. Notable approaches include LDpred [23] and LDpred2 of [16], which compute a polygenic risk score (but not an integrated risk model) by fitting a Bayesian model to given effect sizes via Gibbs sampling. A score is then obtained by inferring the posterior mean effect size of each marker using a prior on the effect sizes and LD information from an external reference panel. Using a normal mixture model offers enhanced flexibility and accuracy through the incorporation of genome-wide markers and different genetic architectures [25,26]. One weakness of Bayesian methods consists in the choice of the required discrete mixture priors on SNP effect sizes, potentially causing computational issues and inaccurate adjustment for local LD patterns.

PRScs of [27] utilizes a high-dimensional Bayesian regression framework which places a continuous shrinkage prior (thus the suffix *CS* for continuous shrinkage) on SNP effect sizes, an innovation which makes a conjugate block update of the SNP effect sizes in posterior inference possible and which is robust to varying genetic architectures.

SBayesR in [26] is a linear regression likelihood which takes into account GWAS summary statistics and a reference LD correlation matrix, and is coupled to a finite mixture of normal priors on the genetic effects. The normal priors allow one to incorporate sparsity and to perform Bayesian posterior inference on the model parameters, such as genetic effects, variance components, and mixing proportions.

The main innovation of MegaPRS [28] consists in the fact that it allows the user to specify how SNPs contribute toward the phenotype. This is done via the specification of a heritability model, which describes how the expected heritability contributed by each SNP varies across the genome. In contrast to current tools which assume that the expected heritability per SNP is constant, the authors show in [28] that realistic heritability models can result in more accurate polygenic risk scores.

Fitting genotype data to a disease outcome can also be achieved by means of a simple penalized regression using the least absolute shrinkage and selection operator (Lasso) in [18], for instance, using the glmnet package on CRAN, see in [29,30]. Glmnet is a fast variant of the FISTA proximal gradient algorithm, the current gold standard for minimizing the Lasso objective function [31]. Glmnet is almost identical to FISTA, but performs a cyclic update of all coordinates, whereas FISTA updates all coordinates per iteration, thus making Glmnet faster than FISTA.

More favorable scaling of polygenic risk score computations (in the size of the input data) has also been a focus in the recent literature [32]. Importantly, machine learning has become increasingly popular for constructing polygenic risk scores [33,34,35,36,37], as machine learning approaches do not assume SNP independence or near independence. However, the resulting prediction model cannot be easily interpreted, in contrast to the linear weighting schemes computed by traditional methods. Examples of traditional approaches outperforming machine learning models are also available in the literature [38].

## 2. Methodology

The Lassosum function of Equation (1) consists of a smooth part, given by $y^{⊤}y+\left(1-s\right)\beta^{⊤}X_{r}^{⊤}X_{r}\beta -2\beta^{⊤}r+s\beta^{⊤}\beta$, and a non-smooth part, the $L_{1}$ penalty ${2\lambda ∥\beta ∥}_{1}$. Only the latter needs smoothing, which we achieve with the help of Nesterov smoothing introduced in Section 2.1. Section 2.2 applies the Nesterov methodology to Lassosum and introduces our proposed smoothed Lassosum objective function. The proposed smoothed Lassosum actually follows from the more general framework of [11,12]. We demonstrate this in Section 2.3, where we also state the theoretical guarantees following from the framework.

### 2.1. Brief Overview of Nesterov Smoothing

In [10], the author introduces a framework to smooth a piecewise affine and convex function $f:R^{q}\to R$, where q∈N. As *f* is piecewise affine, it can be written for $z\in R^{q}$ as

(2) $$\begin{array}{c} f\left(z\right)=\max_{i=1,\ldots ,k}{\left(A{\left[z,1\right]}^{⊤}\right)}_{i}, \end{array}$$

using k∈N linear pieces (components), where $\left[z,1\right]\in R^{q+1}$ denotes the vector obtained by concatenating z and the scalar 1. In Equation (2), the linear coefficients of each of the *k* linear pieces are summarized as a matrix $A\in R^{k\times (q+1)}$ (with the constant coefficients being in column q+1).

The author then introduces a smoothed version of Equation (2) as

(3) $$\begin{array}{c} f^{\mu }\left(z\right)=\max_{w\in Q_{k}}\left\{\langle A{\left[z,1\right]}^{⊤},w\rangle -\mu \rho \left(w\right)\right\}, \end{array}$$

where $Q_{k}=\left\{w\in R^{k}:\sum_{i=1}^{k}w_{i}=1,w_{i}\geq 0\;\forall i=1,\ldots ,k\right\}\subseteq R^{k}$ is the unit simplex in *k* dimensions. The parameter μ≥0 controls the smoothness of the approximation $f^{\mu }$ to *f*, called the Nesterov smoothing parameter. Larger values of μ result in a stronger smoothing effect, while the choice μ=0 recovers $f^{0}=f$. The function ρ is called the proximity function (or prox-function) which is assumed to be non-negative, continuously differentiable, and strongly convex.

Importantly, $f^{\mu }$ is both smooth for any μ>0 and uniformly close to *f*, that is the approximation error is uniformly bounded as

$$\underset{z\in R^{q}}{\sup}\left|f\left(z\right)-f^{\mu }\left(z\right)\right|\leq \mu \underset{w\in Q_{k}}{\sup}\rho \left(w\right)=O\left(\mu \right),$$

see ([10], Theorem 1). Though several choices of the prox-function ρ are considered in [10], we fix one particular choice (called the entropy prox-function) in the remainder of the article for the following reasons: (a) The different prox-functions are equivalent in that all choices yield the same theoretical guarantee and performance and (b) the entropy prox-function leads to a closed-form expression of Equation (3) given by

(4) $$\begin{array}{c} f_{e}^{\mu }\left(z\right)=\mu \log\left(\frac{1}{k}\sum_{i=1}^{k}e^{\frac{{\left(A{\left[z,1\right]}^{⊤}\right)}_{i}}{\mu }}\right), \end{array}$$

which satisfies the uniform bound

(5) $$\begin{array}{c} \underset{z\in R^{q}}{\sup}\left|f\left(z\right)-f_{e}^{\mu }\left(z\right)\right|\leq \mu \log\left(k\right), \end{array}$$

see [10,11,12].

### 2.2. A Smoothed Version of the Lassosum Objective Function

The proposed smoothed Lassosum approach is obtained by applying Nesterov smoothing to the $L_{1}$ penalty of the Lassosum objective function, see Equation (1). A detailed study on the behavior of Nesterov smoothing applied to an $L_{1}$ penalty using synthetic data can be found in [11].

As observed at the beginning of Section 2, it suffices to smooth the non-differentiable penalty ${2\lambda ∥\beta ∥}_{1}$ of the Lassosum objective function, where ${∥\beta ∥}_{1}=\sum_{i=1}^{p}\left|\beta_{i}\right|$. To this end, we apply Nesterov smoothing to each absolute value independently.

We observe that the absolute value can be expressed as piecewise affine function with k=2 components, given by $f\left(z\right)=\max\left\{-z,z\right\}=\max_{i=1,2}{\left(A{\left[z,1\right]}^{⊤}\right)}_{i}$, where

$$A=\left(\begin{array}{cc} -1 & 0\\ 1 & 0 \end{array}\right)$$

and z∈R is a scalar. Substituting this specific choice of *A* into Equation (4) leads to a smoothed approximation of the absolute value given by

(6) $$\begin{array}{c} f_{e}^{\mu }\left(z\right)=\mu \log\left(\frac{1}{2}e^{-z/\mu }+\frac{1}{2}e^{z/\mu }\right). \end{array}$$

Substituting the absolute value in the $L_{1}$ norm in Equation (1) with the approximation in Equation (6) results in a smoothed version of the Lassosum objective function, given by

(7) $$\begin{array}{c} L^{\mu }\left(\beta \right)=y^{⊤}y+\left(1-s\right)\beta^{⊤}X^{⊤}X\beta -2\beta^{⊤}r+s\beta^{⊤}\beta +2\lambda \sum_{i=1}^{p}f_{e}^{\mu }\left(\beta_{i}\right). \end{array}$$

The first derivative of $f_{e}^{\mu }$ is explicitly given by

$$\frac{\partial }{\partial z}f_{e}^{\mu }\left(z\right)=\frac{-e^{-z/\mu }+e^{z/\mu }}{e^{-z/\mu }+e^{z/\mu }}=:g_{e}^{\mu }\left(z\right),$$

see also in [11,12], from which the closed-form gradient of the smoothed Lassosum objective function of Equation (7) immediately follows as

$$\frac{\partial }{\partial \beta }L^{\mu }=\left(1-s\right)2\left(X^{⊤}X\right)\beta -2r+2s\beta +2\lambda \sum_{i=1}^{p}g_{e}^{\mu }\left(\beta_{i}\right).$$

Using the smoothed version of the Lassosum objective function, given by $L^{\mu }$, and its explicit gradient $\frac{\partial }{\partial \beta }L^{\mu }$, an integrated risk model can easily be computed by minimizing $L^{\mu }$ using a quasi-Newton method such as BFGS (Broyden–Fletcher–Goldfarb–Shanno), implemented in the function optim in R [39].

In Equation (7), the quantity *X* is not limited to contain only genotype information. Any data on the individuals (including additional epidemiological covariates) to compute the integrated risk model can be summarized in *X*. The other quantities in Equation (7) are the outcome y (either binary/discrete or continuous), the correlations $r=X^{⊤}y$, and the additional regularization parameter s∈(0,1) introduced in [7] used to ensure stability and uniqueness of the Lasso solution.

### 2.3. Theoretical Guarantees

Using the fact that the absolute value can be expressed as a piecewise affine function with k=2, see Section 2.2, the error bound of Equation (5) can be re-written as

(8) $$\begin{array}{c} \underset{z\in R}{\sup}\left|f\left(z\right)-f_{e}^{\mu }\left(z\right)\right|\leq \mu \log\left(2\right). \end{array}$$

As in our proposed smoothed version of Equation (7) only the non-smooth $L_{1}$ contribution of the original Lassosum objective function of Equation (1) has been replaced, the bound of Equation (8) immediately carries over to a bound on the smoothed Lassosum. In particular,

(9) $$\begin{array}{c} \underset{\beta \in R^{p}}{\sup}\left|L\left(\beta \right)-L_{e}^{\mu }\left(\beta \right)\right|\leq \underset{\beta \in R^{p}}{\sup}2\lambda \left|\sum_{i=1}^{p}\left|\beta_{i}\right|-\sum_{i=1}^{p}g_{e}^{\mu }\left(\beta_{i}\right)\right|\leq 2\lambda p\mu \log\left(2\right). \end{array}$$

For a given computation of an integrated risk model, the Lasso parameter λ>0 and the dimension *p* are fixed by the problem specification. According to Equation (9), this allows one to make the approximation error of our proposed smoothed Lassosum to the original Lassosum arbitrarily small as the smoothing parameter μ→0.

As stated in Section 2.1 in [7], the Lassosum objective of Equation (1) is equivalent to a Lasso problem, in particular its convexity is preserved. According to Proposition 2 in [11], the smooth approximation of Equation (7) obtained via Nesterov smoothing is strictly convex. As strictly convex functions have one unique minimum, and as a closed-form gradient $\frac{\partial }{\partial \beta }L^{\mu }$ of $L^{\mu }$ is available (see Section 2.2), this makes the minimization of our proposed smoothed Lassosum in lieu of the original Lassosum very appealing.

Furthermore, two additional properties of Equation (7) can be derived from ([11], Section 4.3). First, the $\arg\min_{\beta \in R^{p}}L^{\mu }\left(\beta \right)$ is continuous with respect to the supremum norm ([11], Proposition 4), which implies that the minimum of our proposed smoothed Lassosum $L^{\mu }$ converges to the one of the original Lassosum as μ→0. Second, in addition to this qualitative statement, the error between the minimizers of the smoothed and original Lassosum function can be quantified a priori ([11], Proposition 5).

## 3. Application to Experimental Data

In this section, we evaluate the performance of our proposed smoothed Lassosum approach of Section 2.2 in two experimental studies, one fitting an integrated risk model to binary outcomes in the context of Alzheimer’s disease (Section 3.1) using summary statistics, and one fitting an integrated risk model to continuous outcomes using individual-level data in the context of COPD (Section 3.2). We benchmark our smoothed Lassosum approach, which we refer to as “SmoothedLassosum”, against the following state-of-the-art approaches:

1. “Lassosum”: the Lassosum algorithm of [7], implemented in the R package *lassosum* available on github [40].
2. “LDpred2”: the LDpred2 algorithm of [16], implemented in the R package *bigsnpr* on CRAN [41].
3. “PRScs”: the PRScs algorithm of [27], available on github [17].
4. “Glmnet”: the standard lasso of [18], implemented in the *Glmnet* package on CRAN [30].
5. “Lasso”: the unsmoothed Lasso of [12], implemented in the R package *smoothedLasso* on CRAN [42].
6. “SmoothedLasso”: the smoothed Lasso of [12], implemented in the R package *smoothedLasso* on CRAN [42]. Both the unsmoothed and smoothed Lasso are included in the experiments to showcase how the unsmoothed (original) and smoothed Lasso compare.
7. “NeuralNetwork”: a neural network implemented with the *Keras* interface [43] to the *Tensorflow* machine learning platform [44]. We train a network with four layers, having 20, 8, 4 and 2 nodes. We employ the LeakyReLU activation function; a dropout rate of 0.1; a validation splitting rate of 0.1; the *he_normal* truncated normal distribution for kernel initialization; and kernel, bias, and activity regularization with $L_{1}$ penalty. The last layer employs the sigmoid (for Section 3.1) or ReLU (for Section 3.2) activation functions. The model is compiled for binary crossentropy loss (for Section 3.1) or mean absolute error loss (for Section 3.2) using the Adam optimizer, evaluated with the AUC (for Section 3.1) or the mean squared error (for Section 3.2) using 1000 epochs.
8. “SBayesR”: the SBayesR algorithm of [26], implemented in the toolbox *GCTB* [45].
9. “MegaPRS”: we employ the robust version *Bolt Predict* of the MegaPRS algorithm [28] as suggested by the authors. We use default parameters given in the example section of the MegaPRS website (a cross validation proportion of 0.1, the—ignore-weights option and a power parameter of −0.25). MegaPRS is implemented in the *LDAK* package [46].
10. “EpiOnly”: we perform a simple linear regression using epidemiological covariates only.

Unless noted otherwise, all aforementioned methods are run with default parameters. The Lassosum, LDpred2, PRScs, SBayesR, and MegaPRS algorithms are only designed to fit polygenic risk scores, but not integrated risk models. To include epidemiological covariates for these methods (and thus fit an integrated risk model), we first perform a linear regression of the epidemiological covariates to the outcome, and then run the aforementioned methods on the residuals. Importantly, in order to apply Lassosum with epidemiological covariates, we additionally have to recompute the SNP-wise correlation $r=X^{⊤}y$ as in Equation (1) using the residuals in place of y.

Note that Glmnet, as well as Lasso and SmoothedLasso, can be applied in two ways: First, they can be applied to both the epidemiological covariates and genotype information in one go, given all information is summarized in the design matrix. Second, they can likewise be applied to residuals after regressing out all epidemiological covariates. For consistency with the way the Lassosum, LDpred2, PRScs, SBayesR, and MegaPRS algorithms are applied, we also employ Glmnet, Lasso, and SmoothedLasso to residuals after regressing out all epidemiological covariates. Throughout the section, we fix the Lasso regularization parameter at $\lambda =2^{-3}$. This value was chosen in a data-driven way to ensure that the resulting estimates are not too dense (which happens if the regularization parameter is too small), or zero (which happens if the regularization parameter is too large). The Lassosum regularization parameter *s* in Equation (1) (which ensures stability and uniqueness of solution) was chosen as s=0.5 as recommended in Section 3 of [7], and the smoothing parameter of Section 2.2 was chosen as μ=0.1, see Section 3 of [12].

### 3.1. Alzheimer’s Disease Study

We performed training and testing of different PRS algorithms using summary statistics for Alzheimer’s disease (AD), together with genotype data imputed on the Haplotype Reference Consortium (HRC), see in [47]. The HRC-imputed genotype data was downloaded from Partners Biobank [48] (described below). The summary statistics are matched to genotype data for chromosomes 1–22 of 2465 patients available in the Partners Biobank. We considered two sets of summary statistics from two of the largest available AD GWAS: the one of clinically defined AD cases of [13], and the one of AD-by-proxy phenotypes of [14].

The dataset in [13] contains a total of 11,480,632 summary statistics, given by *p*-value, effect size (denoted as variable “Beta”), and standard deviation of the effect size. Each entry is characterized by its chromosome number, position on the chromosome, as well as the effect allele and non-effect allele. The dataset in [14] contains a total of 13,367,299 summary statistics in the same format as the one in [13].

Partners Biobank is a hospital-based cohort from the Mass General Brigham (MGB) hospitals. This cohort includes collected DNA from consented subjects linked to electronic health records. We have obtained a subset in April 2019, which included AD cases and controls. Cases were defined as subjects who were diagnosed with AD based on the International Statistical Classification of Diseases and Related Health Problems (ICD-10), see in [49]. Controls were selected as individuals of age 60 and greater, who had no family history of AD, no diagnosed disease of nervous system (coded as G00-G99 in ICD-10), no mental and behavioral disorders (coded as F01-F99 in ICD-10), and a Charlson Age-Comorbidity Index of 2, 3, or 4 [50,51].

We performed the following quality control steps on the HRC-imputed genotype data from Partners Biobank. Relatedness was assessed with KING [52,53] and population structure was assessed with principal components. Principal components were calculated on a pruned subset (PLINK2 parameters:–indep-pairwise 50 5 0.05) of common variants (MAF >0.1). We excluded subjects which had a KING kinship coefficient > 0.0442 (third degree of relatedness or closer) and which were at least 5 standard deviations away from the mean value of the inbreeding coefficient. We kept only self-reported non-hispanic white (NHW) individuals and excluded outliers, defined as subjects which are at least 5 standard deviations away from the mean value of each of the ten principal components (see Appendix A). There was a total of 2465 subjects (481 cases) left for analysis.

To compare performance across both datasets, we determined the set of variants which are found in both datasets, as well as in the genotype data of the Partners Biobank. We randomly selected 20,000 loci with the–thin-count option in PLINK2 [54]. The precise number of 20,000 loci is arbitrary, and was chosen to include a large number of loci while still being able to run all simulations in reasonable time. Although *APOE* variants are known to have a very high effect size for AD, explaining around a quarter of the total heritability [55], including the *APOE* region in a polygenic risk score or integrated risk model has been shown to be insufficient to account for the large risk attributed to *APOE* [56]. To fine tune our integrated risk models on other *non-APOE* variants with much smaller effect sizes and good prediction power, we decided to keep *APOE* status as a separate predictor. At the same time, we made sure that the extended *APOE* region (from 45,000,000 to 46,000,000 bp on chromosome 19) is excluded while the two *APOE* loci 19:45411941:T:C and 19:45412079:C:T are kept in the data. This leaves 18,038 loci.

**Figure A1:**
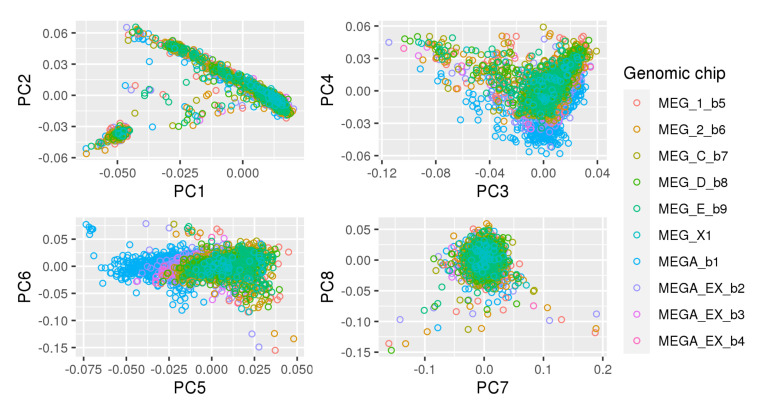
First eight principal components of the HRC-imputed genotype data downloaded from Partners Biobank. Stratification by genomic chip.

The final data used for the computation of the integrated risk models consist of these 18,038 loci, as well as the following epidemiological covariates: age, sex, and *APOE* status with classes “none” (encoded as 0), “single e4” (encoded as 1), or “e4/e4” (encoded as 2). As the data do not exhibit a separation by genomic chip (see Figure A1) we did not include principal components into the model. However, we recommend doing so if a clear separation in the principal component plots is visible.

In the following experiments, we considered the datasets of [13,14] separately and extracted SNP weights based on corresponding effect sizes. This gives us the three quantities X,r,y required to fit the Lassosum model of Equation (1). Next, we only consider a proportion p∈{0.1,…,0.9} of the pool of indices of the Partners genotyped subjects as a training dataset (selected uniformly at random), that is a proportion *p* of the rows of *X* and corresponding entries of y (r is updated using *X* and y) and fit an integrated risk model using Equation (1) to these training subjects with the aforementioned methods. In the case of the neural network, we use the training dataset to tune its hyperparameters. Finally, we evaluate the performance of all methods on the unseen proportion 1−p of the data, that is we compute an estimate of the outcome y with the help of Equation (1) on the unseen data, and compare the outcome estimate to the true outcomes. We report the mean of absolute residuals $\frac{1}{n}\sum_{i=1}^{n}\left|r_{i}\right|$ (where *n* is the number of subjects in the validation set and $r_{i}$ is the residual for subject *i*), the AUC (Area under the ROC Curve), and the correlation between predicted and true outcomes.

**Figure 1:**
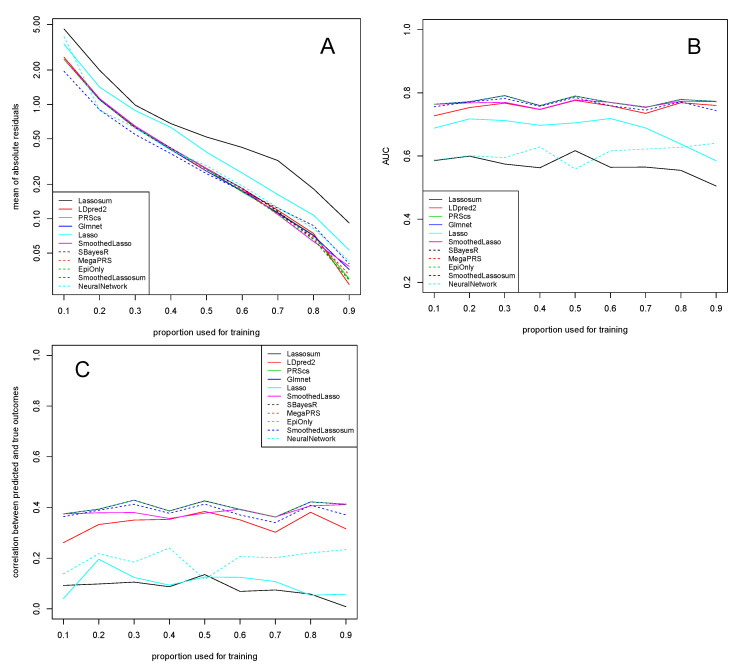
Dataset of clinically defined AD cases of [13]. Mean of absolute residuals (**A**), AUC (**B**), and correlation between predicted and true outcomes (**C**) as a function of the proportion of data used for training. Plotted with jittering.

Figure 1 shows results for the dataset of [13]. A series of observations are noteworthy. First, the mean of absolute residuals decreases with an increasing proportion of the data used for training, as expected.

Second, the AUC is very high (reaching almost 0.80) for all methods apart from Lassosum, Lasso, and NeuralNetwork. Interestingly, it is much less affected than the residuals by the proportion of data used for training and stays essentially constant for all training proportions. This is in line with previous observations that the AUC is invariant to the prior class probabilities [57]. A similar picture is observed when looking at the correlation between predicted and true outcomes, which is roughly equally high for all methods apart from Lassosum, Lasso, and NeuralNetwork. After training, NeuralNetwork achieves a very low mean of absolute residuals, though its AUC and its correlation between predicted and true outcomes somewhat lacks behind the other methods. This is likely a result of the binary cross-entropy loss, which implicitly tunes the behavior towards low residuals. Tensorflow allows for the specification of other loss functions (such as the mean absolute error loss or AUC), though for a binary response the binary cross-entropy loss is a natural choice. NeuralNetwork does manage to achieve an increased performance for higher proportions of training data (in both the AUC metric and with respect to the correlation between predicted and true outcomes). This can be explained with the observation that neural networks typically have many more parameters than conventional methods and thus traditionally require larger amounts of data to be trained on. For instance, the number of nodes per layer, the activation function, dropout rate, etc. per layer all depend on how many layers were chosen in the first place can be freely tuned, thus quickly resulting in large numbers of parameters.

Third, using epidemiological covariates only in a simple linear regression fit seems to perform very well on this dataset. This seems to suggest that actually, the response is well explained by the genetic factor of *APOE* status as well as the other non-genetic factors (such as age), and that the remaining genetic information is rather negligible for prediction.

Fourth, our proposed SmoothedLassosum considerably improves upon Lassosum of [7], now drawing equal with state-of-the-art methodology such as LDpred2 with respect to, e.g., the AUC measure. Moreover, our proposed SmoothedLassosum achieves a considerably improved mean of absolute residuals compared to Lassosum, and a state-of-the-art correlation between predicted and true outcomes. The reason for the reduced performance of Lassosum is not fully understood. However, it is likely related to the fact that Lassosum is not designed to incorporate epidemiological covariates (see Section 4 for more details).

**Figure 2:**
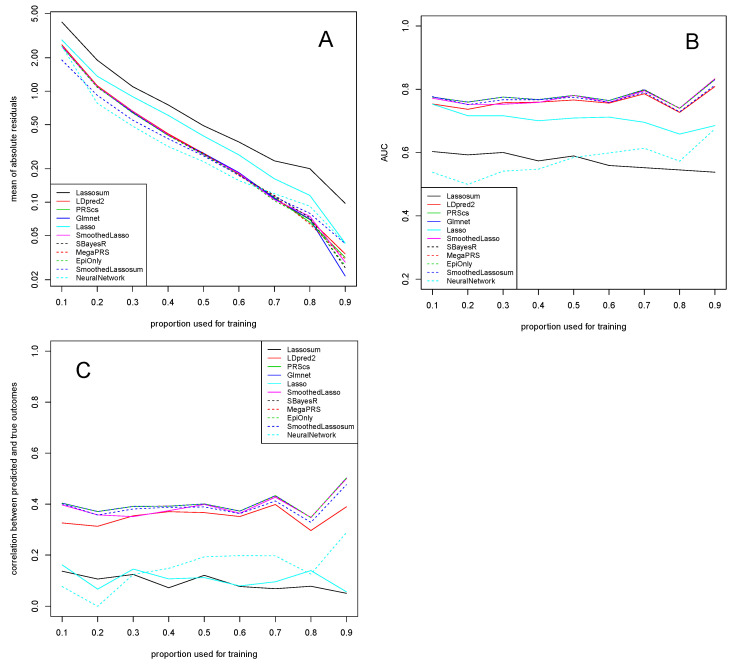
Dataset of AD-by-proxy phenotypes of [14]. Mean of absolute residuals (**A**), AUC (**B**), and correlation between predicted and true outcomes (**C**) as a function of the proportion of data used for training. Plotted with jittering.

The results for the dataset of [14], reported in Figure 2, are almost identical to the ones for the dataset of [13] in Figure 1. In particular, the Lassosum, Lasso, and NeuralNetwork algorithms generally have the weakest performance on this dataset, while the other methods perform equally well. Importantly, SmoothedLassosum considerably improves upon Lassosum by achieving a mean of absolute residuals, AUC, and correlation between predicted and true outcomes that is similar to the others methods.

The similarity between Figure 1 and Figure 2 is expected. The two experiments differ only in the way the response (AD status) is defined. The response provided in [13] consists of clinically defined AD cases, while the one in [14] contains AD-by-proxy phenotypes which are based on 13 independent GWS loci having a strong genetic correlation of (at least) 0.81 with the AD status.

### 3.2. COPD Study

The datasets considered in Section 3.1 are characterized through binary outcomes. In this section, we consider a continuous response in the context of Chronic Obstructive Pulmonary Disease (COPD). To be precise, we look at the COPDGene study in [15], a case–control study of COPD in current and former smokers which has been sequenced as part of the TOPMed Project.

The dataset we consider contains TOPMed WGS data of smokers with COPD, selected as having an age at enrollment of 45–80 years, a smoking history of at least 10 pack-years, non-Hispanic White or non-Hispanic African American descent, and a diagnosis of COPD Stages 2, 3, and 4 by GOLD criteria (post-bronchodilator FEV1/FVC <0.70 and FEV1 < 80% predicted), where FEV1 is defined as the air volume in liters a person can exhale during the first second of a forced expiration, and FEV1/FVC (also called Tiffeneau–Pinelli index) is the proportion of a person’s vital capacity that they are able to expire in the first second of forced expiration (FEV1) to the full forced vital capacity (FVC), see in [58]. We focus on chromosome 15 and consider the risk loci for spirometric measures which have been identified in [59]. Overall, we consider 8881 loci of 3495 individuals.

We aim to predict the raw FEV1 value from the WGS data and four epidemiological covariates, that is, in this section we fit an integrated risk model using individual-level data only. The final data used for the computation of the integrated risk models consists of the raw FEV1 value (the quantity y in Equation (1)) and the 8881 loci plus age, sex, pack-years of smoking, and height (the quantity *X* in Equation (1) from which $r=X^{⊤}y$ can be computed). As in Section 3.1, we use a classic training (proportion p∈(0,1)) and validation (proportion 1−p) setup. Precisely, we only consider a randomly drawn pool of proportion *p* of the rows of *X* and corresponding entries of y for fitting the integrated risk model using Equation (1). After fitting, we compute an estimated outcome by evaluating Equation (1) on the unseen rows of *X* and entries of *y*, allowing us to compare predicted and true outcomes. We apply all algorithms as outlined in Section 3. As the AUC is only defined for a categorical response, we only report the mean of absolute residuals and the correlation between predicted and true outcomes.

**Figure 3:**
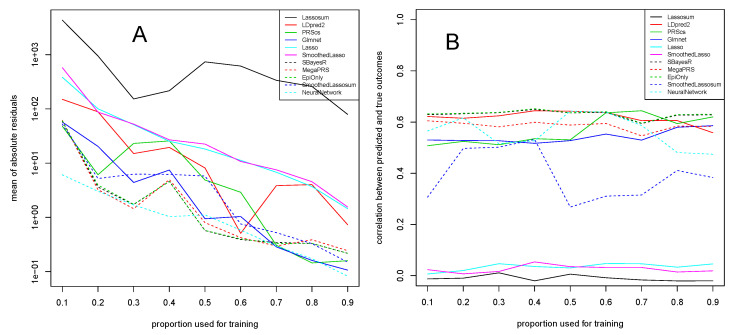
Dataset of the COPD study of [15]. Mean of absolute residuals (**A**) and correlation between predicted and true outcomes (**B**) as a function of the proportion of data used for training.

Results of this experiment are given in Figure 3. We observe that measurements are overall more unstable than in Section 3.1, though as usual, the mean of absolute residuals in Figure 3A decreases with an increasing proportion of the data used for training.

Lassosum is again not performing at its best, which is likely related to the fact that we are aiming to predict a continuous response (see Section 4 for more details). The Lasso and SmoothedLasso approaches are better, showing a good and robust performance throughout all training proportions, although they do not reach the performance of methods such as LDpred2 or PRScs. Together with LDpred2 and PRScs, our proposed SmoothedLassosum approach performs very well and again considerably improves upon the original Lassosum. Glmnet is again one of the best methods together with SBayesR, MegaPRS, though a fit of epidemiological covariates only also seems to have high predictive power. NeuralNetwork seems to be very suited in this experiment to learn the continuous FEV1 responses from the input data.

The correlation between predicted and true outcomes, shown in Figure 3B, confirms that most state-of-the-art algorithms achieve a comparable correlation of around 0.6. The performance of our SmoothedLassosum is slightly worse than those methods with regards to the correlation between predicted and true outcomes, though it again considerably improves upon Lassosum (as well as Lasso and SmoothedLasso) which seem to have difficulties to predict the continuous FEV1 response from this data (see Section 4 for more details).

## 4. Discussion

This article considered the calculation of an integrated risk model by minimizing a smoothed version of the Lassosum objective function (see Equation (1)) introduced in [7]. Utilizing a smoothing approach circumvents the non-differentiability of the $L_{1}$ penalty of Lassosum, thus allowing for an efficient minimization with quasi-Newton algorithms. Our proposed smoothed Lassosum approach can be applied to both summary statistics and individual-level data.

An experimental study on Alzheimer’s disease and COPD demonstrates that our smoothed Lassosum improves upon the original Lassosum of [7], measured with respect to the mean of absolute residuals, the AUC, and the correlation between predicted and true outcomes, thus making it draw equal in accuracy with state-of-the-art approaches (for the datasets under consideration). The reduced performance of Lassosum we observe in the real data applications is likely attributed to the fact that (a) Lassosum is not designed to incorporate epidemiological covariates in integrated risk models, and (b) Lassosum is not designed for continuous responses (as in the COPD study), which occurs, for instance, when regressing out epidemiological covariates and using the residuals as input to Lassosum. In particular, in its original formulation in [7], Lassosum only considers genotype data *X*, and the incorporation of additional covariates is not possible in the Lassosum R package [40]. Moreover, although recomputing the SNP-wise correlation $r=X^{⊤}y$ in Equation (1) and using them in place of y is a valid approach, the distribution of residuals is different from the one of the original binary response (without regressing out the covariates), which might cause a suboptimal behavior of the Lassosum algorithm. For instance, it is not guaranteed any more that each entry of r lays in the open interval (−1,+1) for arbitrary *y*, and it is not straightforward how to transform the input to comply with this condition. In contrast, our smoothed Lassosum works well for both epidemiological covariates and continuous responses.

Using an $L_{1}$ penalty in Equation (1) has the advantage that, in analogy to the original Lasso of [18], computing $\arg\min_{\beta \in R^{p}}L\left(\beta \right)$ performs both regression of the polygenic risk score or integrated risk model and variable selection simultaneously. One potential drawback of our proposed smoothed Lassosum is that it yields dense minimizers (i.e., unused predictors are not necessarily shrunk to zero), meaning that the variable selection property is not preserved. This is not necessarily a disadvantage, as usually the fitted models are only used for risk prediction, for which our dense models achieve a high accuracy. Moreover, other widespread methods such as neural networks likewise do not provide variable selection. If necessary, sparseness can be restored after estimation via thresholding, meaning that all entries $\beta_{i}$ of the estimate β of Equation (1) satisfying $|\beta_{i}|<\tau$ for some threshold τ are set to zero, although this might also cause a decrease in predictive performance [60]. Determining an optimal threshold, as well as the trade-offs incurred compared to working with dense polygenic risk scores, remains for future research.

## 5. Conclusions

We observe that for the prediction of Alzheimer’s disease and COPD, computing an integrated risk model involving genetic information provides little benefit in addition to using epidemiological covariates only. In [61], the authors show that the odds ratio achieved by current polygenic risk scores is too small to warrant their usage as a screening method, and that it would be equally sensible to offer the intervention regardless, given it is effective and inexpensive. In the case of Alzheimer’s disease and COPD, this means that the usage of an integrated risk model is only sensible for costly treatments.

Additional genotype data used in the simulations is available from the Partners Biobank [48]. The summary statistics in [13,14] used in the simulations are available online, see in [62,63].

## Figures and Tables

**Figure A2:**
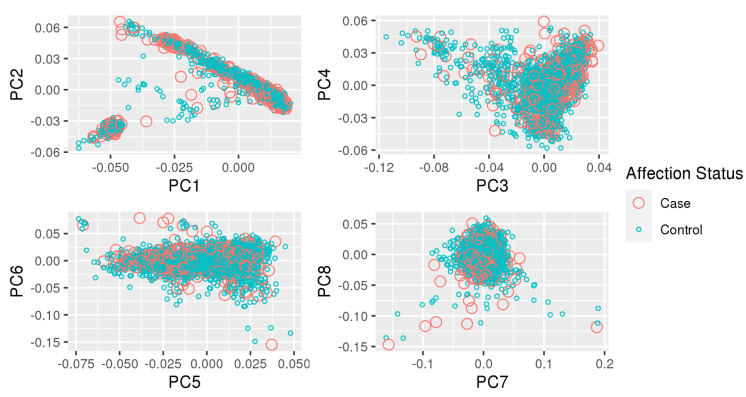
First eight principal components of the HRC-imputed genotype data downloaded from Partners Biobank. Stratification by affection status.

**Figure A3:**
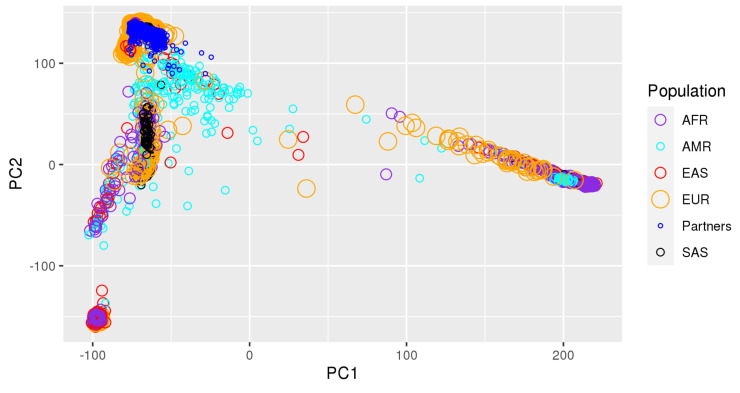
Using *bigsnpr* we have projected our dataset onto the reference 1000 Genomes populations. As the readers can see now, our dataset is clustered within the European population of 1000 Genomes. The population acronyms are: AFR (Africa), AMR (America), EAS (East Asia), EUR (Europe), SAS (South Asian).

## Data Availability

Molecular data for the Trans-Omics in Precision Medicine (TOPMed) program was supported by the National Heart, Lung and Blood Institute (NHLBI). Genome Sequencing for “NHLBI TOPMed: Genetic Epidemiology of COPD (COPDGene) Funded by the National Heart, Lung, and Blood Institute (NHLBI) in the NHLBI Trans-Omics for Precision Medicine (TOPMed) Program” (phs000951.v3.p3) was performed at the Northwest Genomics Center (HHSN268201600032I, 3R01HL089856-08S1, HHSN268201600032I) as well as the Broad Institute Genomics Platform (HHSN 268201500014C, HHSN268201500014C), see Table A1. Core support including centralized genomic read mapping and genotype calling, along with variant quality metrics and filtering were provided by the TOPMed Informatics Research Center (3R01HL-117626-02S1; contract HHSN268201800002I). Core support including phenotype harmonization, data management, sample-identity QC, and general program coordination were provided by the TOPMed Data Coordinating Center (R01HL-120393; U01HL-120393; contract HHSN268201800001I). We gratefully acknowledge the studies and participants who provided biological samples and data for TOPMed.

## Appendix A. Principal Component Plots

Figure A1 and Figure A2 show the first eight principal components of the HRC-imputed genotype data downloaded from Partners Biobank. All individuals we kept in the dataset are self-reported non-Hispanic white (NHW) individuals. We excluded outliers which are at least 5 standard deviations away from the mean value of each of the ten principal components. In Figure A1, we observe a negligible amount of stratification based on the genotyping chip, but given the even distribution of cases/controls across chips displayed in Figure A2; this should not affect the results. Figure A3 shows a projection of our dataset onto the reference 1000 Genomes populations with *bigsnpr*, indicating that our dataset is clustered within the European population of 1000 Genomes.

**Table A1:** TOPMed Omics Support Table. Broad Genomics = Broad Institute Genomics Platform. NWGC = Northwest Genomics Center.

TOPMed	TOPMed Study	TOPMed	TOPMed	Omics Center	Omics Support	Omics Type
Accession #	Short Name	Phase	Project	Short Name		
phs000951	COPDGene	5	COPD	NWGC	HHSN268201600032I	Methylomics
phs000951	COPDGene	2.5	COPD	Broad Genomics	HHSN268201500014C	WGS
phs000951	COPDGene	1	COPD	NWGC	3R01HL089856-08S1	WGS
phs000951	COPDGene	2	COPD	Broad Genomics	HHSN268201500014C	WGS
phs000951	COPDGene	4	COPD	NWGC	HHSN268201600032I	RNASeq
